# Photosynthetic electron transport rate and root dynamics of finger millet in response to *Trichoderma harzianum*

**DOI:** 10.1080/15592324.2022.2146373

**Published:** 2022-11-16

**Authors:** Ramwant Gupta, Munna Singh, Bibi Rafeiza Khan

**Affiliations:** a Department of Biology, Faculty of Natural Sciences, The University of Guyana, Georgetown, Guyana; b G B Pant University of Agriculture and Technology, Pantnagar, India; c Department of Plant Physiology, CBSH, G B Pant University of Agriculture and Technology, Pantnagar, India; d Department of Biology, The University of Scranton, Scranton, PA, USA

**Keywords:** Ragi, photosynthetic electron transport, *trichoderma*, root dynamics, sustainability

## Abstract

Finger millet (ragi) is the main food grain for many people, especially in the arid and semiarid regions of developing countries in Asia and Africa. The grains contain an exceptionally higher amount of Ca (>300 mg/100 g) when compared to other major cereals. For sustainable production of ragi in the current scenario of climate change, this study aimed to evaluate the impact of *Trichoderma harzianum* (TRI) on ragi performance. The performance of photosynthetic pigment pool, photosynthetic apparatus, and root dynamics of three varieties of ragi (PRM-1, PRM-701, and PRM-801) in response to four treatments *viz*., C (soil), S+ TRI (soil + *Trichoderma*), farmyard manure (soil+ FYM), and FYM+TRI (Soil + FYM + *Trichoderma*) were studied. Results have shown a significant increase in the photosynthetic pigment pool and optimized functional and structural integrity of the photosynthetic apparatus in response to the combination of farmyard manure (FYM) with TRI. Higher yield parameters *viz*., φ(Po) and φ(Eo), δ(Ro), efficiency ψ(Eo), performance indices – PI_abs_ and PI_total_, and enhanced root canopy and biomass were observed in all three varieties. Improved electron transport from PSII to PSI, root canopy and biomass, may also suitably favor biological carbon sequestration to retain soil health and plant productivity in case grown in association with FYM and TRI.

## 1. Introduction

One of the minor cereals, finger millet (*Eleusine coracana* (L.) Gaertn), also known as ragi, is a native of Ethiopia and is widely grown in the arid and semiarid regions of developing countries of Asia and Africa.^1–4^ It is a staple food in these nations that provides a significant amount of calories and protein to large portions of the population, especially those in lower socioeconomic groups.^1,5^ Due to its high content in dietary fibers, essential amino acids, iron, zinc, calcium, phosphorus, and potassium, it is nutritionally superior to wheat and rice.^6,7^ Ragi grains contain an exceptionally higher amount of Ca (>300 mg/100 g) when compared to other major cereals.^8^ In the twenty-first century, the world is facing two enormous challenges-first producing more than enough food and fiber to support a growing world population, and second global climate change.^9^ Crop production gains over the previous 50 years have averaged around 1% each year, but in recent decades these rates of increase have halted.^10^ Crop productivity is increased through the breeding of superior varieties, increased application of inorganic fertilizer and agrochemical pesticides, and increased irrigation water utilization. Although that approaches undoubtedly increased global food security, it has reached its limits as yield growth rates have decreased over the previous 20 years.^11^ However, excessive fertilizer use has resulted in major environmental degradation, land contamination, and resource waste.^12^ Recently, a concept of enhanced plant holobionts (EPH) was introduced in which selected fungi and bacteria symbiotically linked colonize the roots of a wide variety of plants, and these plants can enhance photosynthetic capacity by reprogramming gene expression.^13^ *Trichoderma* fungi are free-living organisms that are widespread in the environment. They occur in all climatic zones and colonize different ecological niches. The genus *Trichoderma*’s preferred environment is soil, particularly the rhizosphere of the root system. Numerous metabolites produced by these fungus aid in their interactions with plants and other microbes. It is well known for acting as a growth stimulant and showing up on increased roots and shoots biomass.^9,14^ *Trichoderma harzianum* produces peptides, proteins, and low-molecular-weight compounds that elicit plant defense responses and in addition induces resistance by increasing the expression of defense-related genes throughout the plants.^15^

Induced resistance by Trichoderma spp. increases the expression of defense-related genes throughout the plant, at least in the short term, and is therefore similar to systemic acquired resistance.

Crop yields ultimately depend on the amount of solar light available to plants and the efficiency with which plants convert sunlight into biomass. The energy conversion process occurs in a photosynthetic apparatus located in the thylakoid membrane of the chloroplast. Recently, many attempts had been made to assess the performance of photosynthetic apparatus under several environmental stresses by using chlorophyll a fluorescence OJIP transients.^16,17^ For the O-J-I-P transient, OJ represents the gradual decline of primary electron acceptor quinone (QA) of PSII and JI represents secondary electron acceptor (QB), plastoquinone (PQ), cytochrome (Cyt b_6_f), plastocyanin (PC), and IP is attributed to reduced electron transporters from the PSI ferredoxin (fd), intermediate receptors, and NADP.^18,19^ Quantum efficiency of first-order PSII photochemistry (φPo), the quantum efficiency of electron transport from QA to PQ (ψEo), and quantum efficiency of electron transport from QA to the final PSI acceptor (φRo). The efficiency with which electrons are transferred from PQ to PSI acceptors (δRo) and absorption-based total performance indexes (PIabs) are some additional parameters characterized by O-J-I-P transients.^20,21^

Several studies have been made to understand the photosynthetic efficiency of different plant species in response to *Trichoderma* spp.^9,13,22–26^ However, the response of *Trichoderma* on the functioning of the photosynthetic apparatus in plants has not been elucidated so far. Therefore, the present study is intended to determine efficiency of photosynthetic and root dynamics of ragi in response to *Trichoderma*.

## 2. Materials and methods

Seeds of three varieties of ragi PRM-1, PRM-701, and PRM-801 were obtained from Hybrid Seed Production Center, G.B. Pant University of Agriculture & Technology, Pantnagar, India. *Trichoderma harzianum* (TH38) was obtained from the Center of Advance Study, Department of Plant Pathology, G. B. Pant University of Agriculture & Technology, Pantnagar.

Seeds were grown under greenhouse conditions at G. B. Pant University of Agriculture & Technology, Pantnagar, India, at 29 N latitude and 79 E longitude at an elevation of 243.8 m above the mean sea level. The average temperature for day/night was 30/15°C, the photoperiod for the day-night cycle was 13/11 h, relative humidity 70–75%, and maximum photosynthetically active radiation was about 1100 µmol (photon) m^−2^s^−1^. The experiment was planned using a completely randomized design (CRD) having 3 treatments with 10 replications. The nursery was grown on a tray in sterilized soil, and 21 days old seedlings were transplanted into the 15 cm diameter plastic pots (4 seedlings/pot), filled with 5 kg of sterilized soil, and a mixture of farm yard manure (FYM) with soil in the ratio of 1:1. Four treatments, *viz*., C (soil), S+ TRI (soil + *Trichoderma* @ 0.5 mg/kg), FYM (soil+ FYM), and FYM+TRI (Soil + FYM + *Trichoderma*) were used in this experiment. Data were recorded during vegetative (30 DAT) and reproductive (90 DAT) growth stages. These stages were acquired by the ragi varieties after 51 and 111 days after sowing. The series of observations viz., photosynthetic pigments, OJIP transients, root capacitance, and root biomass were recorded.

### 2.1 Determination of photosynthetic pigments

Chlorophyll was extracted with 80% acetone. The extracts were analyzed with a UV–visible spectrophotometer (Thermo Spectronic Biomate 5) set at 645 and 663 nm and the absorbance of the chlorophyll extract in 100 mL aliquot was recorded. Acetone (80%) solvent was used as a blank. The amount of chlorophyll a, b, and total were determined according to Porra.^25^ Carotenoid content was also measured spectrophotometrically at a wavelength of 480 nm described by Kirk and Allen.^27,28^

### 2.2 Determination of OJIP transients

Chlorophyll a fluorescence transient (OJIP) was measured by Plant Efficiency Analyzer (PEA, Hansatech UK). The clips were placed on the leaves for 20 minutes prior to the measurements to provide dark adaptation. After that the samples were illuminated with continuous red light (wavelength in peak 650 nm, spectral line half-width 22 nm). The light was provided by an array of three light-emitting diodes. The light pulse intensity used was 3500 photons µmol m^−2^ s^−1^, and the duration of the light pulse was 1 s. The fluorescence signal was recorded with a maximum frequency of 100 kHz, after which the frequency of recording gradually decreased collecting a total of 118 points within 1 s ^28^. The measured data were used for the calculation of OJIP transients according to the JIP-test equations.^29,30^

## 2.3 Determination of root capacity

Root capacitance was measured with a BK Precision 810A copper ground rod meter (Maxtech International Corp. Chicago, IL) operating at a frequency of 1 kHz in the range between 200 pF and 2 µF. The electrical contact with the plant was established by connecting the negative electrode to the stem via a battery clamp at 6 cm above level. The positive electrode was connected via a battery clamp to a copper ground rod 55 cm in length inserted into the potting substrate to a depth of 15 cm and positioned 5 cm away from the stem base. The instrument was adjusted at the 20-µF level by setting the readout to zero with the zero-adjust knob. One capacitance measurement per plant was taken at 200µF after allowing 5 s for the system to stabilize (i.e., allowing 5 s for the meter to reach a constant capacitance reading). All capacitance measurements were taken 1 min after watering. Although no standing water was present on the surface of the pots, the soil was fully saturated. The reading was taken at both vegetative and reproductive stages.

### 2.4 Determination of root biomass

For measurement of fresh weight, the plants were harvested, washed with distilled water, and blotted on blotting paper to remove excess water. From each treatment 10 plants were selected. The fresh weight was recorded from the root by using digital single pan balance and values expressed as g/plant. Root length was recorded after harvesting the plants, the values were averaged and expressed as root length in centimeters.

### 2.5 Statistical analysis

The reported data of photosynthetic pigments, i.e. chlorophyll a, b, total chlorophylls and carotenoids, OJIP transients, root capacitance, and root weight represent the standard error of mean in percentage with a 5% value. Statistical analysis was performed using the analysis of variance (ANOVA) followed by the Tukey HSD test (α = 0.05) MS-Excel 2010.

## 3. Results

### 3.1 The photosynthetic pigments

**Figure 1.**
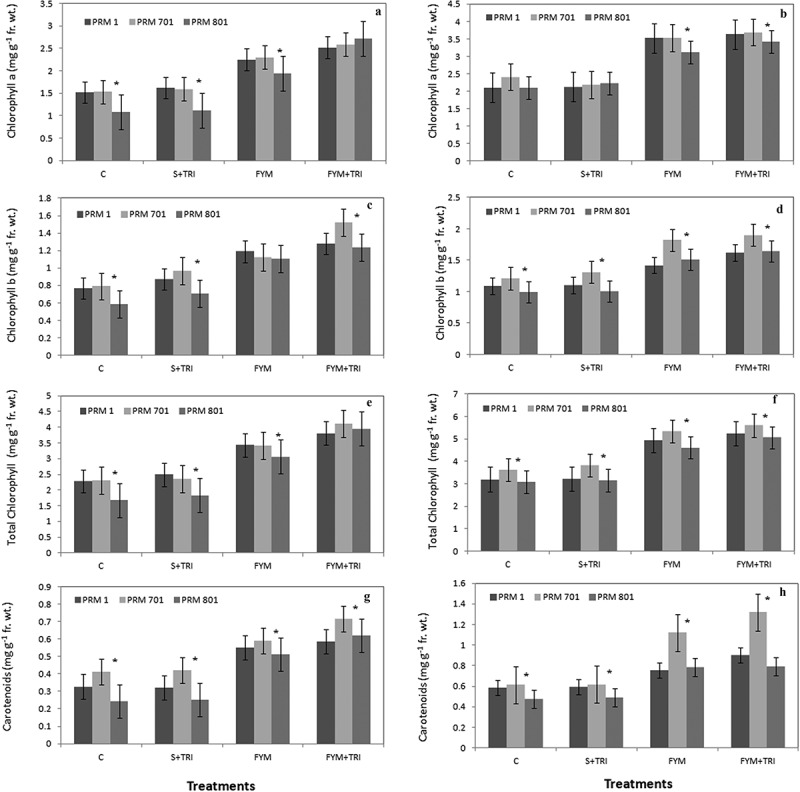
Chlorophyll a (a, b), chlorophyll b (c, d), total chlorophyll (e, f), and carotenoids (g, h) contents of finger millet varieties (PRM-1, PRM-701, and PRM-801) in response to *Trichoderma* at both vegetative and reproductive stages. Each value is the mean of replicates (n = 10) with the standard error of mean in percentage with a 5% value. All three varieties were highly responsive to treatments. Significant differences among the varieties at p < .05 are indicated by Asterisk (Tukey’s test).

The photosynthetic pigments were found to be optimally enhanced in the case treated with farm yard manure in association with *Trichoderma* (FYM+TRI) (Figure 1). Chlorophyll a was almost 2-fold (108%) during the vegetative stage in PRM-801 followed by ca. 75% during the reproductive stage compared to their control (Figure 1, a and b). The other two varieties *i.e*., PRM-701and PRM-1 both showed less of a response with the same treatment in relation to chlorophyll a during vegetative and reproductive growth stages. An enhancement in chlorophyll a content was found ca. 58% and 54% in PRM-1 and PRM-701 during vegetative growth, respectively (Figure 1, a and b). Chlorophyll b data revealed that the optimal was found to be about 2-folds (100%) in PRM-701; however, it was 85% in PRM-801 as compared to control during the vegetative stage (Figure 1c). A similar trend was observed in the reproductive stage with different treatments (Figure 1d). The FYM in combination with *Trichoderma* established superiority in accumulating total chlorophyll content in all three varieties as compared to control in both stages of plant development (Figure 1, e and f). A significant increase in carotenoid content (~90%) was observed in PRM-701 under influence of FYM in combination with *Trichoderma* as compared to control at the vegetative stage (Figure 1g). However, it was increased (~120%) during the reproductive stage (Figure 1h). A similar trend was observed in both PRM-801 and PRM-1 during vegetative and reproductive stages (Figure 1, g and h).

### 3.2 OJIP fluorescence transient

**Figure 2.**
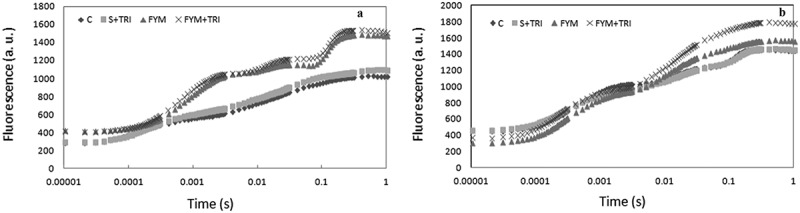
Chlorophyll a fluorescence (a, b) of PRM-701 in response to *Trichoderma* at both vegetative and reproductive stages. OJIP transient curves were induced after different treatments. Here O represents origin (minimal fluorescence Fo), J and I represents the two different inflections (Fj and Fi) and P indicate peak (maximum fluorescence Fp or Fm).

In the present study, to evaluate the photosynthetic function in response to different treatments, the polyphasic chlorophyll transient (OJIP curve indicating the O, J, I, P steps) measurement was examined in the leaves at two different growth stages *viz*., vegetative and reproductive (Figure 2, a and b). After light exposure for 1 s (PEA meter), each step of the OJIP curves exhibited a different response to the different treatments. All four steps O, J, I and P were significantly higher in PRM701 under FYM in combination with *Trichoderma* at both vegetative and reproductive stages. A similar trend was also reflected in response to FYM. On the other hand, the O, J, I, and P steps declined in both soil + TRI and control. The overall results showed a similar pattern of the OJIP curve in both stages; however, the vegetative stage exhibited more fluorescence than the reproductive (Figure 2, a and b).

### 3.4 The maximum quantum efficiency of photosystems

**Figure 3.**
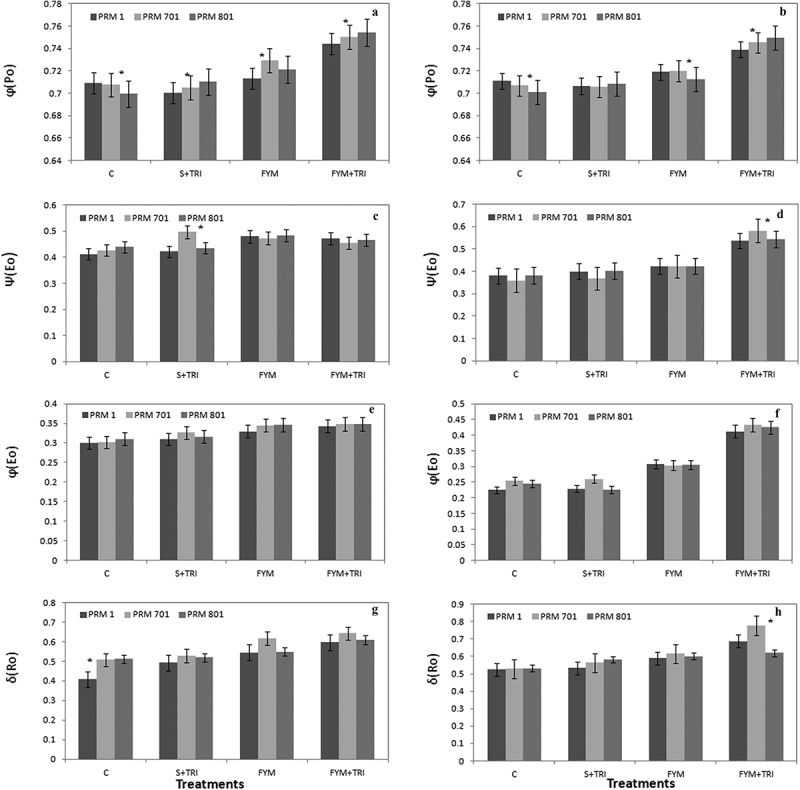
The maximal quantum yield of PSII (φPo) (a, b), the efficiency that an electron moves further than QA (ψ Eo) (c, d); quantum yield of the electron transport flux from QA to QB (φEo) (e, f), and efficiency with which an electron from QB is transferred until PSI acceptors (δ Ro) (g, h) of finger millet varieties (PRM-1, PRM-701, and PRM-801) in response to *Trichoderma* at both vegetative and reproductive stages. Each value is the mean of replicates (n = 10) with the standard error of mean in percentage with a 5% value. All three varieties were highly responsive to treatments. Significant differences among the varieties at p < .05 are indicated by Asterisk (Tukey’s test).

The quantum yield of the Q_A_ reduction (φPo = Fv/Fm) was significantly enhanced in all three varieties at both stages in response to FYM+TRI (Figure 3, a and b). It was an average of 0.70 in the control condition while after treatments were enhanced by 20%, 25%, and 30% in PRM-1, PRM-701 and PRM-801, respectively, under influence of FYM+TRI at the vegetative stage (Figure 3a). The efficiency or probability that a trapped excitation is used for electron transport beyond Q_A_, ψ(Eo) was non-significantly increased in different treatments as compared to control in all three varieties at the vegetative stage (Figure 3c). However, a significantly increased ψ(Eo) ~20%, ~25%, and ~20% in PRM-1, PRM-701, and PRM-801, respectively, was observed under FYM+TRI at the reproductive stage (Figure 3d). The quantum yield of the electron transport from Q_A_, φ(Eo) followed similar trends in all three varieties at the vegetative stage (Figure 3e). However, a non-significant difference was observed at the reproductive stage (figure 3f). The efficiency with which an electron from PQH_2_ is transferred to final PSI acceptors δ(Ro) was significantly increased in all three varieties under FYM+TRI as compared to control. Significantly increased of δRo ~40%, ~50%, and ~40% in PRM-1, PRM-701, and PRM-801, respectively, under FYM+TRI at the reproductive stage (Figure 3, g and h).

**Figure 4.**
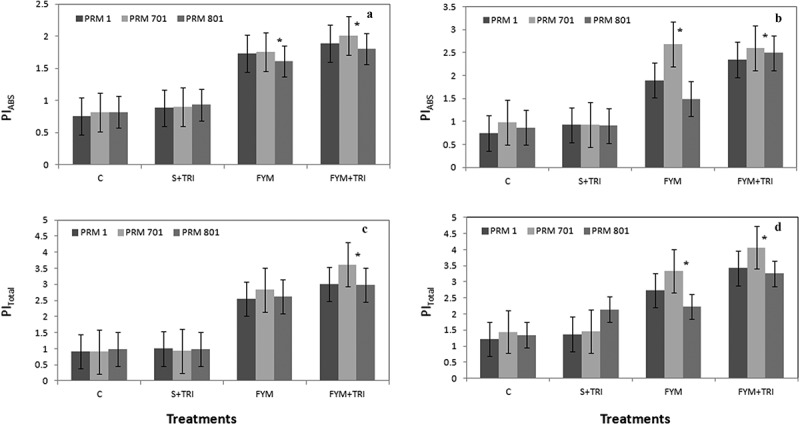
Performance index PI_abs_ (a, b), and PI_total_ (c, d) of finger millet varieties (PRM-1, PRM-701, and PRM-801) in response to *Trichoderma* at both vegetative and reproductive stages. Each value is the mean of replicates (n = 10) with the standard error of mean in percentage with a 5% value. All three varieties were highly responsive to treatments. Significant differences among the varieties at p < .05 are indicated by Asterisk (Tukey’s test).

Performance indices (PIs) are described as combined information on the performance of PSII and reduction of intersystem electron acceptors (PI_abs_) and reduction of PSI end acceptors (PI_Total_). The study revealed that PI_abs_ and PI_Total_ of all three varieties were significantly increased under FYM+TRI treatments as compared to control in both stages. A significant increase of PI_ABS_ in PRM-701 ~ 2-folds and ~3-folds at vegetative and reproductive stages, respectively (Figure 4, a and b). PI _Total_ in PRM-701 ~ 3-folds and ~4 folds at vegetative and reproductive stages, respectively, were reported under FYM+TRI as compared to control (Figure 4, c and d).

### 3.5 Root dynamics

**Figure 5.**
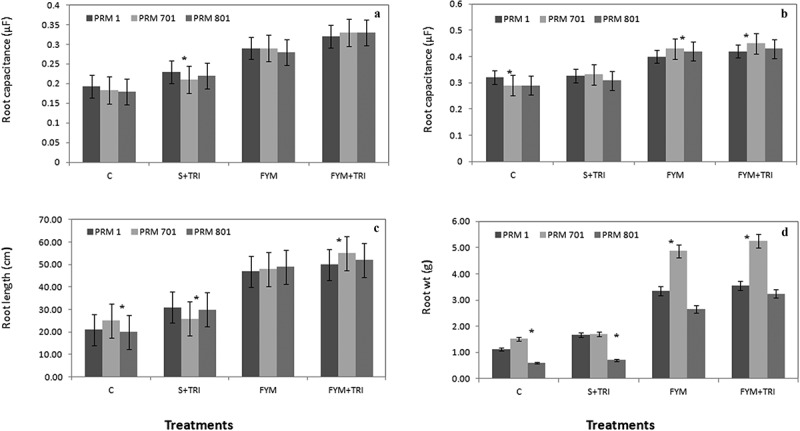
Root capacitance (a, b) at both vegetative and reproductive stages, root length (c), and root wt (d) of finger millet varieties (PRM-1, PRM-701, and PRM-801) in response to *Trichoderma*. Each value is the mean of replicates (n = 10) with the standard error of mean in percentage with a 5% value. All three varieties were highly responsive to treatments. Significant differences among the varieties at p < .05 are indicated by Asterisk (Tukey’s test).

The root capacitance values revealed that the vegetative stage and reproductive stage both dynamically responded to their root capacitance, in assays where plants were given FYM in combination with *Trichoderma*. Root capacitances in PRM-701 of ~45% and ~40% at vegetative and reproductive stages, respectively, were reported under FYM+TRI as compared to control (Figure 5, a and b). A similar trend was observed in all three varieties of ragi in different treatments (Figure 5, a and b).

**Figure 6.**
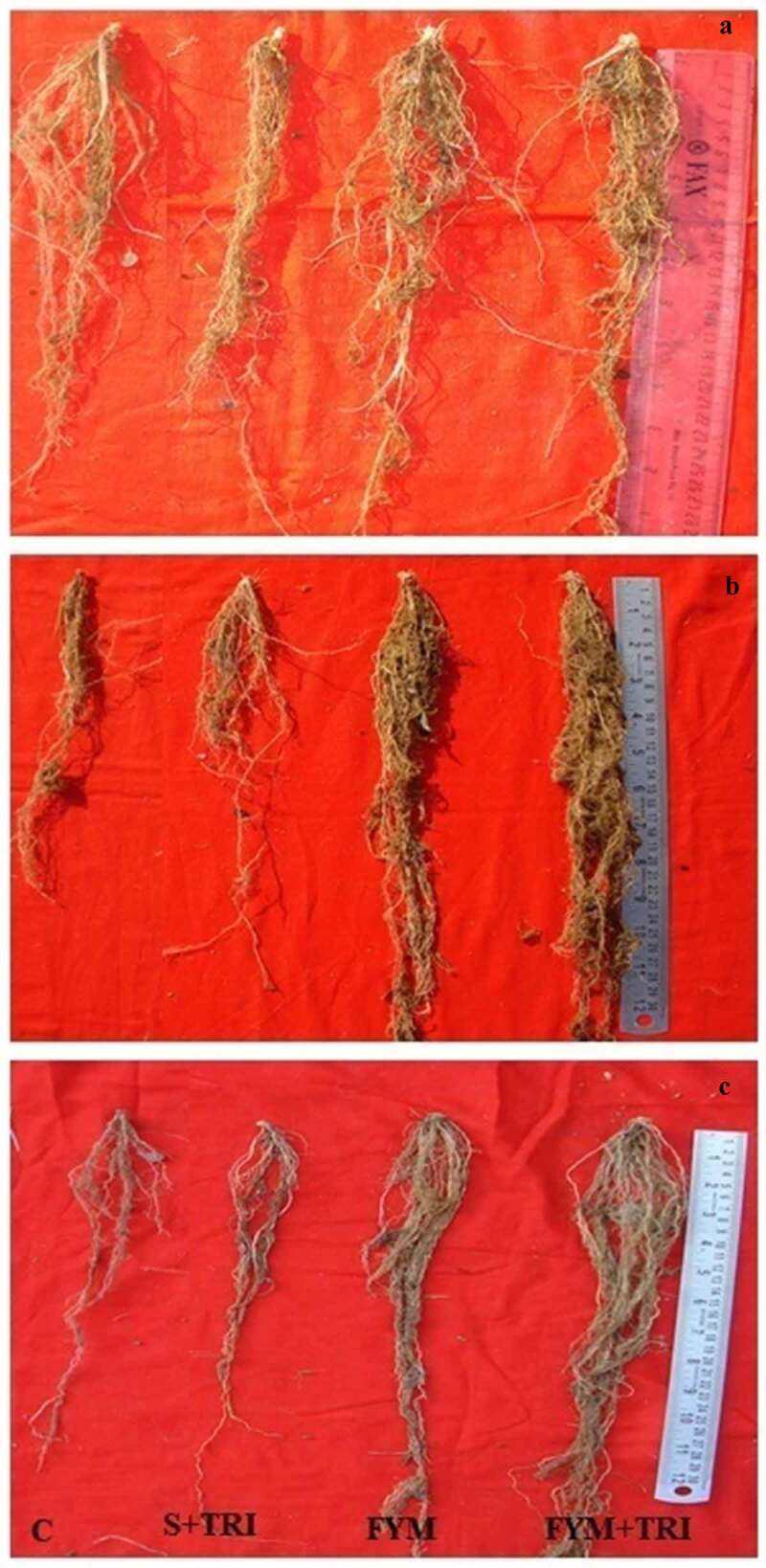
Root morphology of PRM-1 (a), PRM-701(b), and PRM-801(c) in response to different treatments. Root morphology was observed after reproductive stage.

The root biomass was calculated after harvesting ragi varieties. It was found that variety PRM-701 and PRM-801 both have significantly enhanced root biomass. PRM-1 also showed an improved root system of about 2-fold. Root length appears to be positively correlated with the differential application of different treatments (Figure 5c). PRM-701 showed significantly enhanced root length (160%) compared to control. FYM and FYM +TRI treatments were always closely associated with significant root area expansion (Figure 5c). Furthermore, application of FYM and FYM+TRI greatly enhanced root weight (Figure 5d). The percentage increase in the root weight of PRM-701 and PRM- 801 was nearly 4-fold higher in comparison to control (Figure 5d). The morphological appearances of ragi roots shown in Figure 6a–c illustrate the superior root growth upon application of FYM and FYM +TRI. Both treatments Soil + *Trichoderma* and control showed similar trends in root biomass for all three varieties of finger millet (Figure 6a–c).

## 4. Discussion

C_4_ photosynthetic adaptation in finger millet allows it to withstand adverse climatic conditions, high temperature, in particular. Regular use of chemical fertilizers to achieve a better yield, impaired soil health, and may not sustain high crop production even with heavy application of fertilizers. However, biofertilizer application may provide economic and environmental sustainability. This study revealed that treatments with FYM in combination with *Trichoderma* (FYM+TRI) significantly affected the photosynthetic pigment pool. *Trichoderma* is capable of colonizing farmyard manure (FYM) because it serves as an excellent substrate for its multiplication. *Trichoderma* increases the production of growth hormones like IAA.^31^ This phytohormone may account for the root proliferation leading to an increased uptake of water and nutrients eventually resulting in the enhancement of the photosynthetic pigments. In addition, *Trichoderma* increases soil organic matter and essential minerals, such as nitrogen, potassium, phosphorus, magnesium, and the expression of genes regulating the biosynthesis of chlorophyll, which stimulates the development of photosynthetic pigments in plants.^14,31–34^ Many previous studies reported that *Trichoderma* inoculation increased the content of chlorophyll, carotenoids, and total pigments in the leaves of *Allium cepa,^35^ Oryza sativa*,^36^ *Lactuca sativa*,^37^ and *Pistacia vera*.^38^

Chlorophyll fluorescence sign and its calculated parameters have been effectively exploited to investigate and elucidate the performance of photosystems.^18,39,40^ The present findings revealed a significant increase in OJIP transient in ragi under the influence of FYM+TRI at both stages, indicating the optimal performance of the photosynthetic apparatus. To convert light energy into chemical energy, a series of electron transport carriers including PSII, PQ, cytochrome b_6_f, plastocyanin, and PSI are involved.^41^ In this study, a significant increase in φPo (Fv/Fm), ψEo and φEo in ragi varieties under FYM+TRI indicated that the quantum efficiency of PSII photochemistry is increased resulting in speedy electron transport rate.^34^ The efficiency of electron transfers from QB until PSI acceptors (δRo) is optimized in ragi under influence of FYM+TRI, which means the probability that an electron is transported from the reduced intersystem electron acceptor to the final electron acceptor of PSI. Photosystem II oxygen-evolving enzyme and a 13.3-kDa protein (P16.5) similar to thylakoid luminal, which is the center of oxygenic photosynthesis, mediates electron transfer between photosystem II (PSII) and photosystem I (PSI), are upregulated by *Trichoderma* strain in rice.^42^

The performance index on absorption basis PI_abs_ was increased in ragi under FYM+TRI treatment, indicating PSII activity was upregulated. PI_abs_ is produced by a combination of three components RC/ABS (density of active RC per chlorophyll absorption), φPo, and ψEo.^43^ The present study revealed that the intact photosynthetic apparatus in ragi under FYM+TRI led to the speedy energy conversion process. Enhanced PI_total_ under FYM+TRI indicated the improved photosynthetic electron transport activity. Inoculation of *Trichoderma* spp. improved the photosynthetic efficiency in many plants by accumulating photosynthetic pigments, or the expression of genes regulating chlorophyll biosynthesis and proteins of light-harvesting complexes of photosystems.^9^

Better root dynamics were observed under the influence of FYM+TRI in ragi. The phytohormone (IAA) might have been produced by *Trichoderma* to help in the proliferation of roots and thereby increase the uptake of nutrients and a significant increase in the root area, cumulative root length, and root weight over control.^44,45^ The dry weight was also higher in co-cultivated plants, especially in those cultivated with FYM+TRI, similar to *Capsicum annum, Arabidopsis thaliana, Brassica oleracea*, and *Lactuca sativa*.^31,32,46^ The present study provided strong evidence that the electrical capacitance detected between ground and plant electrodes represented the better root system growing in the FYM+TRI.^47,48^ In addition, enhanced plant root growth means that more sequestered carbon will be transferred to roots and stored in the soil.^9^

## 5. Conclusion

*Trichoderma* may enhance food and energy needs for a growing population. Our study explored the higher multiplication efficiency of *Trichoderma* in combination with farm yard manure extended root proliferation to enhance carbon sequestration to retain soil health and its fertility to achieve an eco-friendly rhizosphere. This ensured better root and shoot canopies with improved biological carbon concentrating strategy of ragi plants/crops linked with light harvesting, carbon processing processes, and plant productivity.
